# Liquid Crystal Elastomers—A Path to Biocompatible and Biodegradable 3D-LCE Scaffolds for Tissue Regeneration

**DOI:** 10.3390/ma11030377

**Published:** 2018-03-03

**Authors:** Marianne E. Prévôt, Senay Ustunel, Elda Hegmann

**Affiliations:** 1 Liquid Crystal Institute, Kent State University, Kent, OH 44242, USA; mprevot1@kent.edu (M.E.P.); sustunel@kent.edu (S.U.); 2 Chemical Physics Interdisciplinary Program (CPIP), Kent State University, Kent, OH 44242, USA; 3 Department of Biological Sciences, Kent State University, Kent, OH 44242, USA

**Keywords:** liquid crystals, liquid crystal elastomer, 3D scaffold, biocompatible, biodegradable, tissue engineering, cell alignment, cell proliferation, cell directionality, biomechanics

## Abstract

The development of appropriate materials that can make breakthroughs in tissue engineering has long been pursued by the scientific community. Several types of material have been long tested and re-designed for this purpose. At the same time, liquid crystals (LCs) have captivated the scientific community since their discovery in 1888 and soon after were thought to be, in combination with polymers, artificial muscles. Within the past decade liquid crystal elastomers (LCE) have been attracting increasing interest for their use as smart advanced materials for biological applications. Here, we examine how LCEs can potentially be used as dynamic substrates for culturing cells, moving away from the classical two-dimensional cell-culture nature. We also briefly discuss the integration of a few technologies for the preparation of more sophisticated LCE-composite scaffolds for more dynamic biomaterials. The anisotropic properties of LCEs can be used not only to promote cell attachment and the proliferation of cells, but also to promote cell alignment under LCE-stimulated deformation. 3D LCEs are ideal materials for new insights to simulate and study the development of tissues and the complex interplay between cells.

## 1. Introduction to Liquid Crystal Elastomers (LCEs)

As is well known, several organic substances can exhibit not only solid, liquid and gas phases, they can also show intermediate phases between liquid and solid, which are called mesophases. In 1888, an Australian botanist Friedrich Reinitzer [1] noticed two melting points for cholesterol. Since he could not properly explain this phenomenon, he sent the samples to a German physicist Otto Lehmann [2]. Lehmann, who confirmed the same features found by Reinitzer, explained that they have optical properties identical to a crystal in addition to the flow properties of liquid. Lehmann called these materials liquid crystals (LCs). In crystals, molecules have both positional and orientational order in the given lattice points, contrary to liquid phase. However, liquid-crystalline phase molecules have some degree of orientational and/or sometimes positional order, enabling them to present anisotropy, and also diffuse randomly like liquids [3,4,5]. 

**Figure 1:**
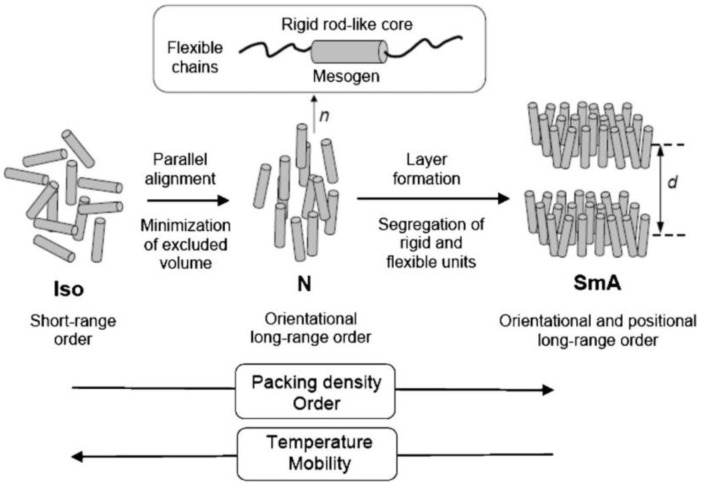
Schematic organization of rod-like molecules in liquid crystal (LC) phases. Reprinted with permission from Reference [4] Copyright 2007 Royal Society of Chemistry.

LC phases are formed by many different types of molecules (called mesogens) such as rod-shaped molecules called calamitic liquid crystals, which is the most common form; disc-like molecules called discotic liquid crystals; and bent-shape (banana shaped) molecules. There are two types of liquid crystal; thermotropic and lyotropic LCs. In thermotropic liquid crystals, mesophases occur by changes in temperature, whereas lyotropic LC mesophases are observed by changes in solution concentration. Moreover, calamitic thermotropic LCs are categorized into two main mesophases; nematic (N) and smectic (Sm). Nematic-phase molecules orient to their preferred direction, which is called the director, and they do not have positional order but long-range directional order. Smectic phase molecules have positional order in addition to directional order and form layered structures, and this phase is divided into different sub-mesophases such as smectic A, B, or C, etc. In smectic A phase, the director is perpendicular to layers, and when this director is not perfectly perpendicular it creates an angle to layers called the Smectic C (SmC) phase (Figure 1). In addition to the structures of these mesophases, LCs can make more sophisticated structures such as the nematic phase of chiral molecules called the cholesteric phase (N*), in which molecules align and have a helical structure. There are also smectic phases formed by chiral molecules, e.g., SmC*. 

**Figure 2:**
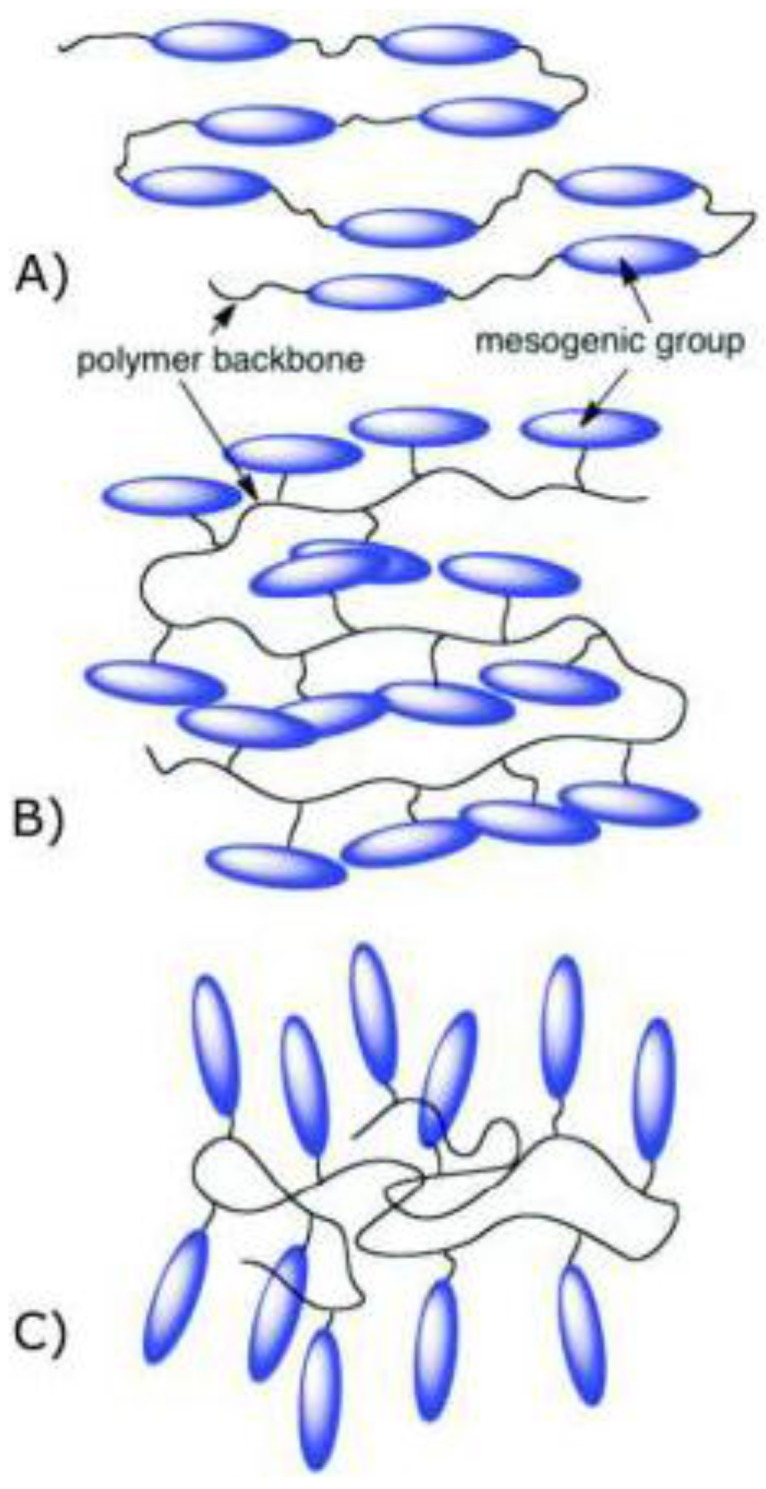
Scheme representation of examples of side-chain and main chain elastomers (**A**) end-on main chain; (**B**) side-on side-chain; and (**C**) end-on side-chain liquid crystal elastomers (LCEs). Reprinted with permission from Reference [11], Copyright 2017 ACS Books.

Polymers are synthetic macromolecules that consist of repeating units of a monomer. Elastomers are also polymers; however, their polymer chains have been crosslinked. The crosslinking density determines the physical properties; thus, elastomers with low crosslinking density have a rubber-like elasticity. During the past decade there has been increasing interest in the synthesis of materials such as liquid crystal polymers (LCPs) that combine the unique properties of low molecular weight LCs with systems containing high molecular weight [6]. Liquid crystal elastomers (LCEs) are polymer-network chains that have a responsive molecular shape and orientational order [7]. Combining the rubber elasticity of elastomers with the directional properties of mesogenic groups [8] (conveying anisotropy to the elastomer), we impart uncommon features to LCEs not shown in traditional non-LC elastomers and polymers. In 1975, de Gennes anticipated the reversibility of their shape property after applying extrinsic stimuli [9], after Lehmann’s suggestion to use the molecular forces of the LC phase for artificial muscles in 1909 [10]. There are two main ways to prepare elastomers: one is where mesogenic units are attached as a side-chain (pendant) to the backbone (mainly in two fashions, see Figure 2) [11], being termed side-chain elastomers; the second is where mesogenic units are attached directly as part of the polymer backbone, termed main-chain elastomers [8,11,12].

Two factors could summarize the LCE synthesis strategies, one being that mesogenic monomers are polymerized and crosslinked together to obtain a network, the other being the use of the side-chain polymer with known phase behavior and then crosslinking. Because of LCEs’ remarkable versatility and functionality, both transition temperatures and the convenience of the network have to be considered during synthesis [13]. The mesogenic groups can also be organized in a way that leads to nematic, smectic, cholesteric forms, among others [14,15]. There are two factors that determine the liquid crystalline phase: the first one is the density of the mesogenic monomers, and the second is translational diffusion and how freely the mesogenic units rotate. In general, the LCEs’ smectic phase is favored by end-on side-chain elastomers, while side-on elastomers prefer the nematic phase because of the long-range positional order [13]. Moreover this orientational order of mesogenic group affects the LCEs’ mechanical behavior. 

As mesogens are chemically bound to the polymer backbone, any change in their orientation will affect the mechanical behavior of the bulk material. The orientation of the mesogenic units is transferred to the whole of the elastomer volume. This network is termed monodomain in contrast to polydomain LCE, where each domain of the material is characterized by a different director. In consequence, monodomain LCEs show macroscopic anisotropic behavior [16]. Polydomain LCEs have similar properties to polycrystalline materials, such as macroscopic isotropy and mechanical deformations giving rise to anisotropy. As reported by Finkelmann et al., nematic polydomain elastomers show a monodomain network when uniaxial deformations convert into macroscopic ordered domains [17]. This phenomenon will be detailed later. Without external stimuli, LCEs mostly show a polydomain network. An additional step is required to align the director throughout the bulk of the LCE, as demonstrated by Fleischmann et al. [18,19], with very recent examples by Yakacki et al. [20,21] and Kim et al. [17,22]. LCEs have different physical properties than standard elastomers due to combination between the network, which has elastic deformation, and the mesogenic monomers’ orientation. Thermal properties are affected numerous variables such as crosslinking density, flexibility of the polymer backbone, type of mesogenic moiety, spacer and degree and type of crosslinking [23]. For example, when crosslinking density increases, the *T_c_* (clearing temperature) decreases. On the other hand, *T_m_* (melting point) and *T_g_* (glass transition temperature) depend mainly on the flexibility of the main chain, and can be relatively easy to increase or decrease [13,24]. While in the past most LC and LCE applications were concentrated on displays, sensors and actuators, in this review we will focus on other interesting applications of LCEs for biological purposes and in particular on tissue engineering. 

We previously discussed the overall interest in using synthetic instead of natural polymers in the tissue-engineering context [11]. However, the limitation regarding non-LC materials concerns their lack of response under a variety of external stimuli and anisotropy. This drawback could be overcome by the incorporation of LC units, which could simultaneously promote dynamic and responsive properties, but also a macroscopic ordering event. The use of LCEs as a muscle-like material is not a new concept, since this idea germinated a century ago. Important highlights in the development of LCEs for biological applications are summarized in Table 1. This timeline will be discussed in the next part.

## 2. Toward Biological Applications of LCEs

**Figure 3:**
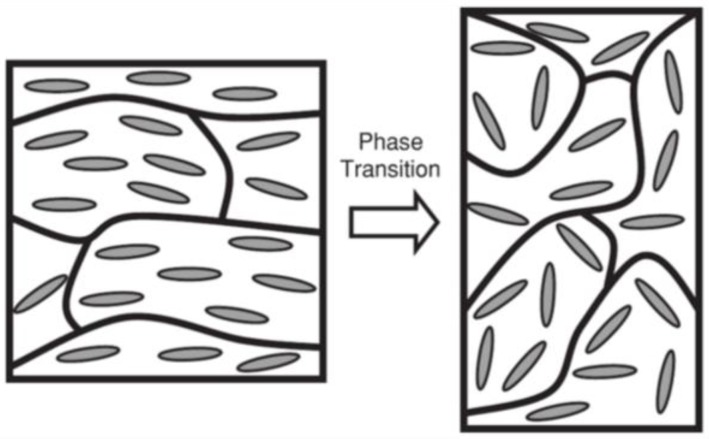
The concept of LCEs as artificial actuator for mimicking muscle. Reprinted with permission from Reference [40]. Copyright 2010, Wiley ACH Advanced Materials.

The field of tissue engineering (TE) first emerged in the 1960s in the wake of the development of a new ‘synthetic substitute for skin’ [33]. This was followed by the first in vitro cultivation of keratinocytes in 1975 [34,35], especially after the release of the first tissue-engineered skin products such as Epicel [36], Apligraf [37] and Dermal Regeneration Template [38]. TE consists in the implantation of a tissue-like structure into the body to repair damaged tissue, injury or a failing organ. Thus, tissue constructs should be carefully chosen to respond to critical functions which can be structural, barrier- and transport-related, or biochemical, and secretory [39]. For LCEs to successfully become a cell scaffold, they need to mimic *in vivo* settings through the control of mechanical, chemical, and geometrical properties. Cell scaffolds should promote the surrounding cell environment to function as native tissue by supporting cell attachment, migration, porous architecture (for the diffusion of nutriments), waste, and mechanical flexibility. LC-based materials have long been considered as possible biological analogues of muscles. The use of liquid crystalline materials in tissue engineering owes its origin to Lehmann who, in a publication named *Liquid Crystal*, published in 1909, defined the concepts of liquid crystals and their applications [10]. By referring to LCs as an artificial muscular rotor, Lehmann initiated the discussion as to whether the liquid crystalline state is essential to life by being a spectacular compromise between order and disorder in living matter. Beyond this speculative idea, de Gennes laid the theoretical foundations of an artificial muscle based on a nematic elastomer: a nematic elastomer across the isotropic (I)-to-LC transition (termed T_LC/I_ in this review) able to cause a strong uniaxial deformation of LCE under external stimulation [9]. This phenomenon was later experimentally confirmed by Finkelmann [26]. The deformations at phase transition manifest themselves locally. To observe the phenomenon on a macroscopic scale, the mesogens are aligned uniformly over the bulk of the material, allowing the direct coupling between the average polymer chain anisotropy and the liquid-crystalline order parameter [40]. As illustrated in Figure 3, in the LC phase (see left side), the polymer backbone shows an anisotropic conformation corresponding to the extended chain arrangement. To be precise, two conformations could be considered, depending on the dimension of the radii of the gyration parallel and perpendicular to the director. In an ellipsoid of revolution, if the rotation is about its major axis, the spheroid conformations are shaped like a rugby ball and called prolate. If the ellipsoid of revolution is rotated about its minor axis, the conformation is a flattened spheroid, known as an oblate. At the phase transition to the isotropic phase (see right side on Figure 3), the anisotropy is lost for a spherical coiled conformation, which leads to the network’s change of dimensions. Thus, samples with oblate conformation expand, while those with prolate polymer chains shrink in the direction of the director, and the change of the order of parameter is induced by a temperature variation. Over these two decades, significant progress has been made in the actuation of LCEs for biological applications becoming what is today a promising multidisciplinary area of research.

Finally, the goal of tissue engineering consists in: (i) the development of a human-made actuator material which generates a large mechanical deformation induced by an external stimulus; (ii) the secretion by the cells of their own support structure, called the extracellular matrix (ECM), which allow cells to respond to signals and organize into tissue. This part of the review gives an overview of the advances in TE using LCEs as actuators. Then, we will report on the latest research undertaken on LCEs and cells.

### 2.1. Synthesis of Monodomain LCEs and Their Mechanical Characteristics

Several methods to synthesize LCE have been reported. For nematic LCEs, the Finkelmann method was the first developed. It describes a two-step crosslinking process, the first being slight crosslinking of LCPs, followed by the orientation of the mesogenic groups; the LCP is then stretched and crosslinked to maintain the orientation [26]. This technique was also applied to other liquid-crystalline phases (e.g., cholesteric, smectic) [41,42,43,44]. Other methods have been reported to prepare LCE with different properties, such as reversible crosslinking by a trans-esterification reaction [45], coupling reactions of thiol-acrylate used for the production of LCEs and aligned by stretching [20] or by means of patterned surfaces [46], and a thiolyne coupling reaction developed to produce aligned LCEs using direct light alignment (DLW) surfaces [47]. Others have used photo-induced thiolene chemistry to produce micron-sized LCE pillars [48] or produce voxelated LCEs by photo-induced crosslinking (following Michael’s addition reactions) [49]. Kularatne et al. categorized these in two main groups by at least one polymeric precursor used and synthesized from monomeric precursors [12]. Also, Ohm et al. categorized the different synthetic pathways to prepare LCEs [40]: the first being Finkelmann’s method, which relates to mixing all ingredients (mesogens, crosslinker and polyhydrosiloxane chain) in a two-step palladium-catalyzed reaction; the second is where, under certain conditions, crosslinker reacts with the functional groups of the LC polymer by mixing them [40]. The third synthetic pathway is the activation of crosslinking groups that are already contained in LC polymer. The last is where a crosslinker and liquid-crystalline monomer are mixed and polymerized [40]. 

The mechanical properties of LCEs were predicted by de Gennes as presenting a non-linear stress-strain relation [9]. The flexible backbone of LCEs enables reorientation of mesogens that can be triggered by external stimuli (e.g., temperature, mechanical stress, electric and magnetic fields, etc.) giving rise to transferring stress and strain via the backbone to ensure mechanical work. As mentioned, the reorientation of mesogenic groups in LCEs can produce monodomain LCEs. Stress (*σ*)-strain (*ε*) measurements increase by raising the applied strain, and the plateau of this measurement corresponds to the transition of polydomain to monodomain [50]. The mechanical properties of a LCE are dependent on temperature and time above the critical temperature [51]. Kock et al. observed a dramatic change above the critical temperature in stress measurement [17].

### 2.2. Actuation Properties of LCEs

The magnitude of deformation of monodomain LCE with temperature is governed by the coupling between the liquid-crystalline state, the linking of mesogens, and the polymer chains. In the case of nematic main-chain polymers, large conformational changes are observed, while only rigid rod-like mesogenic side-chains are coupled to the chain conformation in nematic side-chain polymers, leading to a smaller deformation [52]. Another impact concerns the nature of the LC phase; while larger changes are expected in the most ordered smectic arrangement, no clear examples of this hypothesis have emerged, as we will report later.

The functionality of the actuation of LCE is limited to a defined temperature range, determined by the chemical constitution of the polymer networks. However, since the mechanism of actuation is the phase-transition switching, any external source that could activate such temperature transition can therefore be used to trigger the actuation. Thus, for sensitizing LCEs to an external energy source, the incorporation of stimuli-responsive organic functional groups could be considered. These will be considered within the following sub-sections.

#### 2.2.1. Thermo-Responsive LCEs

In 1993, Warner et al. predicted the “soft elastic response” in pure monodomain nematic side-chain LCE [53], corresponding to a non-elastic energy cost or spontaneous anisotropy of the rubbery network in polymer backbones, coupled to mesogenic moieties that align and form the nematic order [54]. In 2001, Wermter and Finkelmann demonstrated than nematic siloxane-based networks with LC main-chain polymers improve this ability considerably, giving them efficiency as thermally stimulated mechanical actuators [29]. Due to the direct coupling of the LC main-chain segments to the network anisotropy, elongation of up to 40% is observed when concentration is increased. In the same vein, Clarke et al. synthesized side-chain siloxane polymer monodomain nematic LCE siloxane-based films. They studied the dependence of sensitive functions with the way networks are crosslinked [55]. As noticed by Finkelmann, increasing the proportion of main-chain nematic polymer in the side-chain nematic network has a significant impact on the average effective anisotropy of polymer chains, leading to a shape change of 300%. Based on the same idea, and with the same compounds, a deformation of over 300% was also achieved by Tajbakhsh et al. [54]. The challenge for this kind of system to become a real muscle model lies in the reduction of the transition temperatures, as T_LC/I_ was found from 80 °C to over 100 °C in the previously discussed examples. In 2008, Bispo and Finkelmann proposed modifying the nematic siloxane-based monomer core of the main-chain LCE by changing the symmetry of the mesogen through methylation of the central ring in order to promote room-temperature activity of these actuators [56]. Another limitation is the incorporation of the highly anisotropic main-chain, causing a slow relaxation time. Thus, increasing the amount of the main chain in a side-chain-containing nematic network induces a larger magnitude of spontaneous strain. However, this addition implies a slowing down of the response properties. De Gennes predicted that this limitation concerning the response time derives from heat diffusion in the order of seconds for a size of 0.1 mm [23]. According to his suspicions, engineering a new set-up could surpass this limitation. This point will be discussed later in the incorporation of innovative technologies in LCE-scaffold formation. 

**Figure 4:**
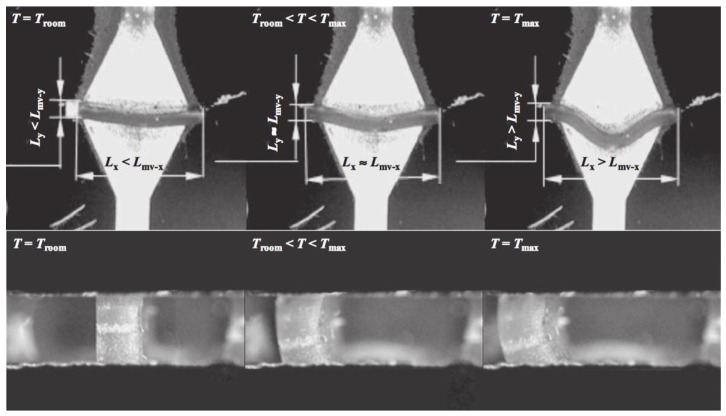
Snapshots pictures taken during the actuation of the LCE microvalve developed by Sanchez-Ferrer et al. The working temperature is T_N/I_ = 80.5 °C. Reprinted with permission from Reference [57]. Copyright 2011, Wiley ACH Advanced Materials.

Besides the development of main-chain LCEs, some teams have worked on this thermomechanical effect in side-on nematic LCEs. Thomsen et al. showed a strain change of 35–45% through a mixture of acryloyl benzoates [28]. They found the strongest coupling when the length of the spacer between the liquid-crystalline mesogen and the polymer backbone is short. In 2011, Sanchez-Ferrez et al. presented the integration of monodomain nematic side-chain LCE into a silicon-based microstructured device, working as a microvalve [57]. The expansion of the LCE in the directions perpendicular to the director has been found at a maximum of 120%, while the shrinkage in the direction parallel to the director shows a 69% change (see Figure 4).

#### 2.2.2. Photo-Responsive LCEs

**Figure 5:**
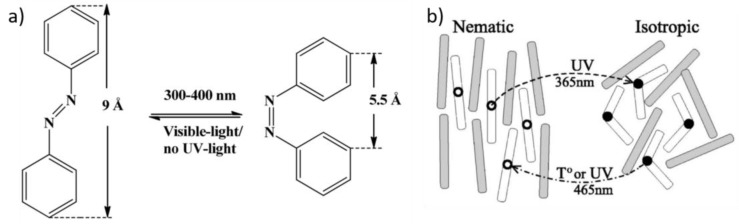
(**a**) The *trans*-*cis* isomerization of azobenzene, reprinted with permission from Reference [63]. Copyright 2013, MDPI Materials; (**b**) schematic depiction of nematic-isotropic phase transformation in a LC containing photo-isomerizable mesogenic molecules, which turn from a rod-like *trans* to a kinked *cis* conformation under ultraviolet (UV) irradiation. Reprinted with permission from Reference [64]. Copyright 2002, APS Physical Reviews E.

Several photoresponsive LCEs have been developed by introducing photochromic groups into the LCEs. Most of these photochromic molecules are azobenzene derivatives, characterized by –N=N– linkage as functional groups in the polymeric architecture. An azo compound is represented by a core molecular structure formed by two moieties bonded to the azo bridging group. Properties depend on the azo linkage and the groups on both sides of the bridge and, specifically, many aromatic azo compounds have the ability to absorb visible light (400–700 nm). Photoresponsive behavior of azo elastomers are caused by the photoisomerization of aromatic azo groups. Azobenzenes possess two isomeric configurations: a thermally stable *trans* state and a meta-stable *cis* form, triggered under irradiation (Figure 5a). When the system is irradiated with a suitable wavelength of light (approximatively 365 nm), the *trans* azobenzenes will be efficiently converted to the *cis* form, consequently reducing the molecular size [16], as illustrated in Figure 5a. The rod-like shape stabilizes the liquid-crystalline phase, whereas the bent shape acts as a non-mesogenic impurity, implying a decrease of the nematic order and a shift of the clearing point to lower temperatures (Figure 5b). This process is photo-reversible by switching off ultraviolet (UV) light or by irradiating with visible light (465 nm) [58]. Here, the remarkable work of Ikeda et al. should be acknowledged [16,59,60,61,62].

The previous examples show conversion in polydomain LCE, leading to no preferential direction for deformation. Hence, the degree of deformation of the material is generally small and requires polarized light. A spontaneous contraction along the director will take place in monodomain LCEs from the ordered liquid-crystalline state to the isotropic state by the action of heat. 

**Figure 6:**
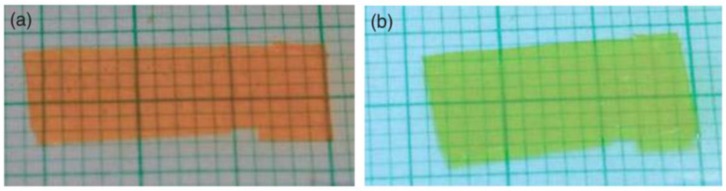
Contraction in a monodomain side-on nematic azo LCE film between (**a**) initial state (before irradiation); and (**b**) under UV irradiation (background in graduated paper). Reprinted with permission from Reference [65], Copyright 2003, Wiley ACH, Advanced Materials.

Liquid crystallinity within an azo-based elastomer could be induced either by the intrinsic mesogenic nature of azobenzene moieties and/or through copolymerization with monomers with mesogenic groups. Finkelmann and coworkers first succeeded in inducing a contraction by about 20% in an azobenzene-containing monodomain side-chain LCE [30]. This material suffered from its slow dynamics on both sides in the order of minutes, even hours under irradiation. In 2002, Li et al. surpassed this drawback by demonstrating a monodomain side-chain nematic azo LCE which exhibits between 12% and 18% contraction in the film in approximatively 1 min (see Figure 6) [65]. The actuation speed seems to be dominated by the photoisomerisation dynamics, which could be increased by using more intense radiation [58].

The introduction of photo-sensitive azoderivatives in monodomain LCEs, not only as side-chain groups but as cross-linkers as well, induces the sample’s control of the macroscopic uniaxial dimensions isothermally just by applying light [58,64]. A 35% expansion was obtained with reduced temperature of the experiment (around 60 °C) in the work of Hogan et al. [64]. Both examples, characteristic “on” and “off” working ranges, strongly depend on temperature. However, it is generally found to be very long, and the working temperatures are still above physiological conditions. From these two studies, it appears that photomechanical deformation is governed by the proportion and position of azobenzene moieties. Azo-containing LCEs have inherent limitations related to the penetration of light within the material. To achieve contraction of the LCE rather than a bend, the film needs to be thin and exposed to light over a long period to permit the light to penetrate the bulk of the material [66].

#### 2.2.3. Summary

The above displays the potential of LCEs for mechanical dynamic substrate in muscle reconstruction, as stimuli-responsive materials with reversible and large-amplitude shape-response. Muscles exhibit contraction/elongation along the fiber axis. Three variables need to be taken account to mimic a muscle: the deformation strain, the stress that can be exerted, and the contraction frequency. Concerning the first variable, it is known that the viability of cells cannot be ensured beyond 30% strain. Thus, this strain defines a requirement in our field of research. Table 2 summarizes the main characteristics of thermo- and photo-responsive LCE films reported in this review. We were interested in comparing the performances of the main systems made for tissue reconstruction and to understand why, despite their potential, LCEs are still rarely used as implants. Table 2 shows that the limitations involve the fact that the transition temperatures T_CL/I_ are typically higher than physiological conditions which are a requirement to ensure the viability of cells on the LCE surface. Moreover, the systems described report mechanical properties without a target tissue in mind. However, our approach consists in the development of a synthetical pathway permitting the tuning of mechanical properties to mimic a specific cell’s environment. In this context, attention should be given to the work of McKee et al. [67] who clearly underlined the effect of the measurement method on mechanical results. Another constraint that we emphasized in the previous paragraph is the slow shape-response time of current actuators. Electrically activated LCEs could have much faster response speeds (in the order of ms); Lehmann et al. have also shown a 4% strain at 133 Hz [68]. These performances limit LCEs’ ability to compete as artificial muscles. Such applications require materials that are biocompatible and that can respond quickly to mild stimuli, such as magnetic fields or small changes in temperature. Additionally, there is a concern about the use of toxic moieties, especially azo derivatives for light-responsive materials. The next part will be dedicated to improvements on these two main limitations by the incorporation of other technologies. 

### 2.3. Integration of Other Technologies for the Preparation of Refined LCE Biomaterials and Scaffolds for Tissue Transplants

The incorporation of a myriad of technologies is key to yielding complex prototype tissue substitutes [11]. Technologies such as particle leaching, emulsification, gas foaming, nanofiber self-assembly, electrospinning, 3D printing, soft lithography, microfluidics, and bioreactors have greatly impacted TE. In this review, we will discuss some of examples of new generation of LCE-based scaffold-meeting technologies. 

#### 2.3.1. Development of Sophisticated Scaffolds

As highlighted in our previous work, an ideal scaffold should satisfy certain requirements including a porous 3D architecture that allows cell growth, vascularization and transport of nutrients between the cells seeded within the matrix and the surroundings [4,11]. To become the support of high cell-seeding density and unimpeded tissue growth, this structure should show well-interconnected open-pore architecture. 

Particle leaching consists in the dispersion of a porogen into the pre-LCE solution. The polymer–porogen network is then solidified, and the solute particles are then subsequently leached, or dissolved away. The result is a porous structure with regular porosity controlled by the size of the leached/dissolved particle. For orthopedic applications, Yakacki et al. tailored porous structures based on the salt-leaching method [69], which we also recently replicated for the formation of LCE-based foams [70]. Targeting brain tissue reconstruction, we engineered the size of the leaching particles to allow a co-culturing of nerve cells. 

Additionally, porous architectures can be created by forming an emulsion, i.e., mixing a solution of LCE with water. We succeeded in making a microemulsion photopolymerization resulting in nematic LCE microspheres (10−30 μm) [71,72].

Microfabrication and microelectromechanical systems (MEMS) techniques, can help reach the size scale, and controlled features for a more complex *in vivo* environment [73]. Buguin and Keller created micron-sized responsive pillars based on monodomain nematic LCE using the soft lithography technique [74]. Not long after, by the same procedure, they managed to create other patterns (large and small cylindrical pillars, and square), and their thiolene nematic main-chain LCEs showed a large contraction of around 300–400% [48]. By photolithography, Elias et al. developed shaped surfaces by producing microscale devices anchored on a substrate [75]. 

Some progress has been made in the preparation of artificial muscle-fiber architecture to mimic natural muscle-fiber bundles. Electrospinning appears a useful technique to produce continuous fibers from nanometer to submicrometer diameters and to create 3D-porous scaffold with any desirable pore size. For example, Krause and Finkelmann succeeded in obtaining mechanical fiber actuators of about 450 nm diameter based on a main-chain LCE with a uniform alignment of the nematic director [76]. Another technique developed by Naciri et al. draws the fibers by dipping a metallic tweezer and pulling fibers out of the mixture [77]. In turn, Ahir et al. produced fibers by a micro-compounding technique drawing a roll of long fiber of diameter ~0.2 mm [78].

3D-printing technology represents an advanced technique in the proposal of precise and complex manufacturing, and could, as such, be convenient for designing muscle tissue. A large number of 3D-printing scaffolds have already been imagined for tissue repair [79]. Recently, Yakacki et al. combined 3D printing and LCEs for the fabrication of a soft actuator [80]. Ware et al. have printed thermally responsive LCEs into 3D structures, where each aligned LCE filament undergoes 40% reversible contraction along the print direction on heating [81]. In January 2018, C&EN news magazine highlighted the work of Ozbolat [82] on the use of a 3D-printing scaffold to improve mechanical and cell adhesion properties compared to the conventional cast method [83].

Another challenge consists in manipulating fluid and cells in geometries of submillimeter scale. Zentel and collaborators counted on the use of a microfluidic device to prepare droplets of LCE material showing a monodomain alignment of the mesogens with the flow field of the macrofluidic setup [84]. On particles for which the size was comprised between 280 mm and 550 mm, they observed a 70% length change [84]. Based on this idea, microscale monodomain colloidal actuators have been fabricated [85].

#### 2.3.2. Development of Composite LCE Biomaterials 

Deformation of LCE systems is achieved by heating the material at the temperature of phase change. However, the low thermal and electrical conductivity of polymeric LCEs implies an inefficient coupling of external heat into the film, leading to a low response time. Embedding nanoparticles into LCEs provides a strategy for adding functionality to LCEs and ultimately enhancing their performance, thereby improving their control over the actuation. The thermal coupling is improved by doping the LCE film with an appropriate conducting material. In such structures, heat could also be coupled into the film by Joule heating in the form of an electrical current throughout the film. More detailed information could be found in the 2012 review of Tenrentjev and collaborators [66].

Different kinds of nanocomponents have been proposed for incorporation within the LCE network, such as carbon black [86] and carbon nanotubes [32,87]. For instance, Courty and Terentjev demonstrated that only a low concentration of carbon nanotubes (in the order of 0.01%) creates a large effective dielectric anisotropy of the composite, giving the composite the potential to be an electrically driven actuator [32]. Finkelmann et al. have demonstrated that the incorporation of super-paramagnetite nanoparticles show a fast local temperature rise that leads to the contraction at the nematic-isotropic transition with short response time (several seconds) [52]. Shenoy et al. used a surface layer approach to the control of LCEs using electrical stimuli, namely they coated the LCE surface with a thin layer of conducting carbon. They showed improvement in actuation time without losing mechanical properties of the LCE film [88].

The techniques and examples mentioned are summarized in Table 3.

## 3. From Cell-Cell to LCE-Cell Interactions 

The previous part has shown that the collective molecular behavior of LC materials could mimic the dynamic functioning of muscles. Now, we will demonstrate that LC-based materials should match requirements for the attachment and proliferation of cells, and could be used to exert control over mechanical cell behavior. Extracellular matrix (ECM) consists of proteins and macromolecules secreted by cells to create a meshwork and provide structural support. ECM connects surrounding cells, providing biochemical support, which is fundamental for cellular activities such as differentiation, proliferation, migration etc. [89,90]. Cell alignment, which refers to the spatial and oriented organization of cells, plays a critical role in ECM remodeling, and in the modulation of mechanical properties for some tissues [91]. Thus, the engineering of cell alignment needs to be considered as a critical point to reach TE.

LCs are omnipresent in biological systems. As is known, LCs are categorized in two groups; thermotropic and lyotropic liquid crystals. Lyotropic liquid crystals are obtained at certain temperature and at suitable concentration by the dispersion of amphiphilic compounds in a solvent. Living cells were described as LCs in 1933 by Bernal [92]. Brown in 1979 wrote a book titled *Liquid Crystals and Biological Structures* to explain liquid-crystalline systems in cells such as the cellular membrane, polypeptide solutions, lipids etc. [93,94,95] Also, it has been shown that short DNA and RNA oligomers may have LC phases [96,97] and Clark, et al. showed that LC ordering is also found in randomly sequenced DNA, ultrashort DNA oligomers, and double stranded DNA [98,99]. In his review, *Liquid Crystal and life*, Goodby insists that DNA could exhibit mesomorphic behavior, and phospholipids, which are a class of lipids present in cell membranes that have lyotropic and thermotropic LC behaviors [100].

The biocompatibility of materials is mainly determined by their capability to co-exist within body tissues without producing any harm or damage. Biomaterials should be designed to mainly be non-toxic and non-carcinogenic, in other words, to not promote any immunogenic or inflammatory response [11,101]. Efforts have been devoted on identifying non-toxic LCs for cell applications. Luk et al. have worked on the interface between mammalian cells and thermotropic LCs [102]. LCEs soon after being prepared should be tested for cell viability, proliferation, and cytotoxicity. Cells should be allowed to attach and proliferate for at least 24 h prior conducting any viability test. We have previously shown with PrestoBlue (cell viability), CyQuant (cell proliferation), and Cyto Tox-Fluor (cytotoxicity assay) that all our as prepared LCE scaffolds unmistakably supported cellular viability and proliferation without inherent cytotoxicity [11,71,103,104]. Yakacki et al. have focused their attention on the development of a new method to synthesize and program permanently aligned monodomain LCEs [20]. They proved that LCE networks have a cytocompatibility response at both stages of the reaction. Agrawal and collaborators reported colloid-in-liquid-crystal gels suitable for the adhesion and proliferation of mammalian cells [105].

Performing biodegradability tests on LCEs is an important key for demonstrating their biodegradable nature, as well as for determining the degradation rates. Prior to designing a LCE it is important to take into account the selection of every component and their ability to biodegrade. In our case, we selected to work with lactones-based elastomers, caprolactone and/or lactide monomers because of their known capability to degrade primarily via bulk acid-catalyzed hydrolysis resulting in non-toxic six-carbon fragments [11,106]. We performed biodegradability studies using slab specimens of several LCE compositions, and studied against non-LC elastomers, using phosphate-buffered saline (PBS) at different pH values (3, 7, and 11) or cell culture media. Slab samples are usually examined by weighing for a period of at least eight weeks and following % weight [104,107], and/or mechanical changes of the samples as a function of time. LCEs and non-LC elastomer samples mostly are expected, most of the time, to demonstrate a main increase of weight in the first week due to water absorption (swelling). [70,104,108] Weight initially and during the degradation studies increases, since the formation of degradation products within the matrix draws water into the polymer matrix via osmosis. Soon afterwards, the resulting degradation products will leak out of the elastomer/polymer slab and start the osmotic process once again. We have, however, not found many similar biodegradable studies for LCEs designed for their use as cell scaffolds. 

Once the coexistence of cells and LC/LCE materials was proven to be compatible, some studies announced the impact for cell growth within liquid-crystalline ordering. For example, Fang et al. demonstrated that 5CB LC could assume distinct orientations when it was layered over several different cell lines cultured on glass substrates [109]. Lockwood and Abbott looked at thick films of TL205 LC coated with a thin film of Matrigel [110]. They first noticed that the device could support the growth of human embryonic stem cells (hESC), but also that the reorganization of the Matrigel over time by the hESCs could generate an orientation transition within the TL205 film. This particular organization of LC could become an easily optically discernable response by engineering the device using a polarized light. Similar biomolecular and/or mechanical interactions of cells and LCs have been observed at single covered colloidal micrometer LC domains [105].

It is, therefore, clear that LC units could be used as dynamic material for cell growth. Nevertheless, this dynamical signaling needs to be seen from the opposite point of view, that of the cells. We should externally stimulate the LCEs (producing a new anisotropy or conformation at the molecular level); this stimulation will provide an input to the cells. Cells, for their part, will act and posteriorly react to LCE, creating an output that will potentially affect the anisotropy of the LCEs and be optically detected. The expectation in cell culturing is to create a dynamic substrate exploiting the ordering of the LCs to promote, direct, and report anisotropic cell growth. In 2009, Kirkwood and coworkers presented collagen films characterized by a cholesteric fibril structure with controlled organization [111]. They observed that human fibroblasts cultured on these substrates have anisotropic growth in their long axes along the direction of the banding. This study constitutes the first evidence of the fact that liquid-crystalline architecture provides a contact guiding signal to the cells. Two years later, Lai et al. introduced oriented fibrils in anisotropic collagen, leading also to the directional growth and elongation of fibroblasts [112].

**Figure 7:**
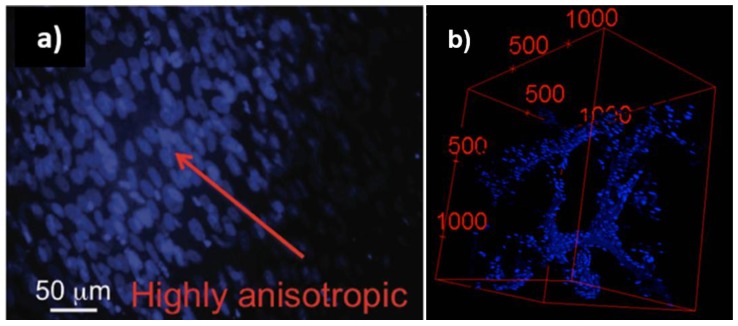
(**a**) Primary human dermal fibroblast (hDF) cultures grown for 5 days on 4LCE-α, grown in film LCE films, reprinted with permission from Reference [103], Copyright 2017 WILEY-VCH Macromolecular Bioscience; and (**b**) fluorescence confocal microscopy images of myoblast cells (C2C12) cultured in LCE foam stained with DAPI (for cell nuclei), 2D images stacked in z direction. Scale units are in μm. Reprinted with permission from Reference [118] Copyright 2016, ACS Macro Letters.

Inspired by these results, our team succeeded in creating the first proof-of-concept use of LCEs as porous film-cell culture scaffold [104]. The mechanical properties were adjusted to seed the side-chain smectic LCEs with cholesteric LC moieties with skeletal muscle cells. Human myoblasts (C2C12) were grown for about 1 month in 60 and 150 μm-thick elastomer films. These preliminary results were followed by the study of cellular responses to LC units [103]. In addition to the C2C12 cell line, human dermal fibroblasts (hDF) cells were considered. Confocal microscopy not only has been a useful tool for following cell proliferation [11,71,103,104,113] within the bulk of LCEs, it also allows for the study of cell behavior such as cell alignment [103]. In the particular case of cell alignment, we demonstrated that by staining only the cell nuclei we observed an extended cell nuclei (cellular response to substrate strain [114] and/or topographical cues [115]). The cell nuclei alignment and its particular deviation from a spherical shape were analyzed using ImageJ [116,117]. Beyond the growth, expansion and proliferation of cells in both cases, we showed a remarkably anisotropic directional cell growth without any external stimulus applied, indicating that cells sense the lamellar molecular structure of the LC components embedded within the scaffold network (Figure 7a).

We then pursued our research by seeding 3D-interconnected open-pores structures. As mentioned, to promote a better attachment, as well as cell infiltration, vascularization and transport of nutriments between the cells seeded within the matrix and the surroundings, a highly porous 3D scaffold is required. Two kinds of morphologies were investigated: globular and foam microstructures. Based on a microemulsion photopolymerization method, we obtained nematic LCE microspheres corresponding to a globular morphology with internal pores formed by the voids between the spheres [71]. In this structure, with LCE particles (10−25 μm in diameter) cells were found to have an adhesion directly onto the globular surface, growth after 7 days, and proliferation within the bulk of the globular morphology. This study highlights the fact that the surface roughness and the porosity improve the attachment of cells and their permeation into deeper regions of the scaffold. Nematic globular LCEs were also found to be excellent scaffolds for different cell lines such as C2C12, hDF and neuroblastoma (SHSY5Y). Besides permeation, expansion was observed for SHSY5Y by the presence of morphological phenotypes indicative of matured cells, namely neuritic extensions. The second morphology we focused on concerned a highly regular foam-like architecture based on a nickel strut template (Figure 7b) [118]. This template is etched to create a foam inside the LCE. This structure contains a primary porosity characterized by spatially interlaced, interconnected microchannels and, depending on the application, could include an additional secondary porosity featuring microchannel networks. This dual porosity aims to mimic the macroscale morphology of the vascular networks observed in tissue. This study allows us to validate the use of elastomeric material considering that 3D elastomer foams show four-times higher cell-proliferation capability compared to conventional porous templated films. Additionally, the same tendency of spontaneous cell alignment than that in porous film has been observed in the 3D structure, leading to a cell orientation in parallel within straight-channel sections, and is retained in curved-channel sections. This alignment tendency of the cells along channels is clearly indicated by elongated nuclei. C2C12 cells grown on a LCE foam scaffold thrive in the more open 3D microenvironment compared to a 2D control (where cells commonly grow on top of one another fighting for space), and cells interact freely with neighboring cells without facing any spatial constraints. 

More generally, we proposed the synthesis of a smectic side-chain LCE containing different kinds of lactone and lactide, in which the initiator, the crosslinking degree, and the position of the LC pendants are all parameters we can adjust depending on the tissue target to tune the mechanical properties and biodegradability [119,120,121]. A broad range of cells have exhibited affinity with the above LCE structures, such as hDF primary cell lines, SH-SY5Y, and C2C12 cells. However, other examples of cells that can be cultivated include, but are not limited to, endothelial, stem cells, brain (glial, neurons, etc.), liver (hepatocyte), red blood types (erythrocyte), bone (osteocytes), skin (keratinocyte), muscle (myocite) cells, and any other somatic cells. Recently, we have shifted our interest toward a new method of creating LCE-based foams with a highly interconnected open-pore structure and tunable morphology. Indeed, cell size varies from tissue to tissue and a suitable pore-size is required to allow cell infiltration and transport of nutrients [11]. We succeeded in the realization of smectic side-chain LCE-based foams made with a particle-leaching method, which exhibit adaptable mechanical, biodegradable, biochemical as well as geometrical properties [70]. 

Agrawal and Verduzco created a thermo-responsive monodomain polysiloxane-based side-group LCE, which exhibits fast uniaxial elongation and contraction when immersed in an aqueous medium on top of resistive heaters [122]. The contraction is engineered to require a low level of heat, supplied locally to the LCE, and provide significant bulk temperature changes and macroscopic shape-changes. For the study, the LCE substrates were subjected to cyclic heating that produced uniaxial strains of up to 5% in contact with water. They observed, under cyclic stimulation, an alignment of the cardiomyocytes along the primary direction of strain. This work constitutes the first example of stimuli-responsive LCE substrates for dynamic cell culture. In the design reported, the heating element is directly in contact with the substrate. Thereafter, the authors proposed a device containing carbon nanoparticles to activate cell cultures [123]. They implemented these biocompatible LCE nanocomposites, capable of a fast and large cyclic, electromechanical actuation as substrates for myocyte culturing. Samples were subjected to a 3% electromechanical strain, myocytes were viable, and the samples showed high cell attachment. This work opens the path of conductive substrates for the generation of responsive LCEs reacting to a broad range of stimuli such as magnetic fields or light. Herrera-Posada et al., for their part, prepared a monodomain magnetic-sensitized polysiloxane-based LCE with iron oxide nanoparticles [124]. First, the addition of nanoparticles reduces the nematic-to-isotropic phase transition temperature, which allows an actuation of the LCEs at physiological temperature. Second, NIH-3T3 fibroblasts attached, and proliferate on LCE dynamic substrates; the embedded magnetic particles did not have a negative effect on the survival of the cells. Recently, Martella et al. were interested in seeding C2C12 and hDF cell lines into an acrylate-based LCE with homogeneous planar alignment [113]. In this monodomain texture, cells tend to orient and differentiate, and transversal cell–cell contact is generated. However, Martella et al. highlight that there is no proof that cells’ orientation corresponds to the unidirectional alignment of the LC order. A work on light-responsive structured LCE for in situ temporal control of surface properties to guide cell migration has been realized by Koçer et al. [125]. These azobenzene-based systems can be programmed to present photo-switchable micrometer-scale topographies and differences in surface roughness. Upon in situ temporal changes in surface nano-roughness, cell migration patterns are switching, with a tunable mobility. This work leads to the possibility of guiding cells to specific locations on the surface.

## 4. Summary and LCEs’ Future Challenges in Tissue Engineering

The story of LCs started in 1888 and since then they have long been considered a fascinating material that responds to external stimuli. LCEs were sought as artificial tissues due to the combination of orientational ordering, induced by LC moieties, and the elastic properties of polymers. While LCs and LCEs appeared promising for biological applications, most of their applications were focused on displays, so interest in biomaterials stayed dormant for decades. An awakening to the use of LCs and LCEs as biomaterials started at the turn of the 21st century. Since then, several research groups have been working intensely to apply the knowledge of LCs/LCEs to the design of new LC materials for the study of biological systems. Despite their potential, LCEs still have some limitations to overcome, mainly related to having transition temperatures within physiological conditions (to ensure the viability of cells and implanted tissue), the tuning of mechanical properties to mimic specific native environments, and faster response times. Most synthesized LCEs that have been tested for in vitro viability, proliferation and cytotoxicity and have proven to be fully biocompatible and allow for cell studies. However, until now, no *in vivo* tests have yet been performed or reported. Only non-LC elastomers have been used and tested in vivo [39,126]. Nevertheless, since LCEs are made of biocompatible materials similar to those of non-LC elastomers, their introduction and use for *in vivo* systems does not appear to be problematic. We believe that this review summarizes some of the key properties of LCEs, which make them an ideal class of materials that can bring breakthroughs in tissue engineering. LCEs support reporting, directing and influencing the behavior of biological systems in a two-way communication for a better understanding of the complex interplay between cells and biological systems. 

## 5. Patents

1. A. Neshat, A. Sharma, T. Hegmann, and E. Hegmann, 04/15/2013, US PCT 61/853993 “Biodegradable side-chain LC elastomers: smart responsive scaffolds”
2. Y. Gao, T. Hegmann, and E. Hegmann, 12/19/2014; PCT/US2014/071618 “Biocompatible: smart responsive scaffold having interconnected pores”
3. M. Prévôt, T. Hegmann, and E. Hegmann, Filing of Conversion Application claiming priority to US 62/902, filed 10/16/2017; Biodegradable, biocompatible 3D liquid crystal elastomeric foam scaffolds having tailor-made animal (human) pore cells sizes via a salt leaching method are capable of growing tissue therein for therapeutic reconstruction of damaged and/or diseased tissue or organs.

## Figures and Tables

**Table 1:** Timeline of LCE studies leading towards biological applications.

Actors	Year of Publication	Events
Lehmann [10]	1909	Allusion to artifical muscular motor based on LCE system.
DeGennes [9]	1975	Theoritical analysis of the dynamic and the thermodynamic properties of Nematic LCE. Theoritical foundations of artificial muscle-based on contraction of LCE at T_N/I_.
DeGennes [25]	1982	Evidence that polymer chains can elongate in nematic phase.
Kupfer and Finkelmann [26]	1991	Experimental confirmation of DeGennes’ work in monodomain nematic LCE.
Li and Keller [27]	1994	Polymer chains can recover a random coil conformation change at isotropic phase.
Thomsen and Keller [28] Wermter and Finkelmann [29]	2001	Thermo-responsive muscle-like materials.
Finkelmann [30] Li and Keller [31]	2001 2003	Photo-responsive muscle-like material.
Courty and Tenretjev [32]	2003	Electro-responsive muscle-like material.

**Table 2:** Overview of the main characteristics of thermo- and photo-responsive LCE films reported in this review.

Contributors	Type	Monomer	Elongation (%)	T_LC/I_ (°C)	Young Modulus
Wermter and Finkelmann 2001 [29]	Nematic Side-Chain + Main-Chain	Siloxane	40	96	16 MPa
Clark and Terentjev 2001 [54]	Nematic Side-Chain	Siloxane	300	107	2–200 kPa
Tajbakhsh and Terentjev 2001 [54]	Nematic Side-Chain	Siloxane	300	85–108	30 kPa
Bispo and Finkelmann 2008 [56]	Nematic Main-Chain	Siloxane	30–35	Room Temperature	56 MPa
Thomsen and Ratna 2001 [28]	Nematic Side-Chain	Acryloyl benzoate	35–45	85–120	210–270 kPa
Sanchez-Ferrer and Finkelmann 2011 [57]	Nematic Side-Chain	Butyl benzoate	69 (shrinkage) 120 (expansion)	80	65–80 kPa
Finkelmann 2001 [30]	Nematic Side-Chain	Azo-containing silylene	10–400	40–50	-
Li and Keller 2003 [31]	Nematic Side-Chain	Azo-containing Methacrylate	12–18	86–90	210 kPa
Hogan and Tenretjev 2002 [64]	Nematic Side-Chain	Azo-containing Siloxane	35	57–110	-

**Table 3:** Overview of technologies and techniques used to implement geometries and actuation properties of LCE scaffolds for tissue engineering.

Technology/Technique	Features	Examples of Scaffold
Particle leaching	3D porous structures with regular and controlled porosity given by the porogen’s characteristics	DiRienzo and Yakacki [69] Prévôt and Hegmann [70]
Emulsion	3D porous architecture with any pore size	Bera and Hegmann [71]
Micro-electro-mechanical systems (MEMS)	3D porous complex structures for micro-actuation	Buguin and Keller [74] Elias and Broer [75]
Electrospinning	3D porous scaffold mimicking muscles from nanometer to submicrometer diameters	Krause and Finkelmann [76] Naciri and Keller [77] Ahir and Terentjev [78]
3D printing	Precise and complex 3D porous design	Yuan and Yakacki [80] Ambulo and Ware [81]
Macrofluidic	3D scaffold suitable for fluids and suspended cells in submillimeter scale	Ohm and Zentel [84] Marshall and Terentjev [85]
Incorporation of nanoparticles	Increasing of the actuation properties of scaffold	Chambers and Finkelmann [86] Courty and Terentjev [32] Landi and Gennett [87] Shenoy and Keller [88] Kaiser and Finkelmann [52]
